# Use of Principal Component Analysis to Combine Genetic Merit for Heat Stress and for Fat and Protein Yield in Spanish Autochthonous Dairy Goat Breeds

**DOI:** 10.3390/ani11030736

**Published:** 2021-03-08

**Authors:** Alberto Menéndez-Buxadera, Eva Muñoz-Mejías, Manuel Sánchez, Juan Manuel Serradilla, Antonio Molina

**Affiliations:** 1 Meragem Group, Department of Genetic, Campus de Rabanales, University of Córdoba, Ctra, Madrid-Cádiz, km 396, 14014 Córdoba, Spain; contact@ambuxadera.com (A.M.-B.); pa1semaj@uco.es (J.M.S.); 2 Department of Animal Pathology, Animal Production, Food Science and Food Technology, University of Las Palmas de Gran Canaria, 35413 Las Palmas, Spain; gerente@gescansl.com; 3 Department of Animal Production, Campus de Rabanales, University of Córdoba, Ctra, Madrid-Cádiz, km 396, 14014 Córdoba, Spain; pa1sarom@uco.es

**Keywords:** dairy goat, heat stress, random regression model

## Abstract

**Simple Summary:**

Tolerance to heat stress (HS) is an important economic trait in goats to maintain dairy farm profitability. We studied the effect of a climatological index of temperature and relative humidity (THI) on test day record (TD) of fat and protein yield (fpy) in the three main Spanish dairy goat breeds (Florida, 126.825 TD, Malagueña, 141.856 TD and Murciano Granadina, 62.834 TD), analysing the nature of the fpy response throughout the THI on the weeks of lactation (DIM) trajectories. The results showed the existence of a double genotype environment interaction between DIM and THI and a depression in the fpy level in animals kept in the hot zone (THI > 25) compared with those in the cold zone (THI ≤ 16). This negative impact is equivalent to 13 to 30 days in production. We propose an alternative to select animals by using a principal analysis of the estimated breeding value for each goat breed across DIM and THI scales. The results of this study show that this procedure is a powerful tool to select the most productive and heat-tolerant animals, thus contributing to the increased profitability of autochthonous Spanish goat breeds.

**Abstract:**

We studied the effect of the Temperature Humidity Index (THI) (i.e., the average of temperature and relative humidity registered at meteorological stations) closest to the farms taken during the test day (TD), for total daily protein and fat yields (fpy) of the three main Spanish dairy goats. The data were from Florida (11,244 animals and 126,825 TD), Malagueña (12,215 animals and 141,856 TD) and Murciano Granadina (5162 animals and 62,834 TD) breeding programs and were studied by different linear models to estimate the nature of the fpy response throughout the THI and the weeks of lactation (Days in Milk, DIM) trajectories. The results showed an antagonism between THI and DIM, with a marked depression in the fpy level in animals kept in the hot zone of the THI values (THI > 25) compared with those in the cold zone (THI ≤ 16), with a negative impact equivalent to production of 13 to 30 days. We used a Reaction Norm model (RN), including THI and DIM as fixed covariates and a Test Day Model (TDM), to estimate the genetic (co)variance components. The heritability and genetic correlations estimated with RN and TDM showed a decreased pattern along the scale of THI and DIM, with slight differences between breeds, meaning that there was significant genetic variability in the animal’s ability to react to different levels of THI, which is not constant throughout the DIM, showing the existence of genotype-environment interaction. The breeding values (BV) of all animals for each level of THI and DIM were subject to a principal component analysis, and the results showed that 89 to 98% of the variance between the BV was explained by the two first eigenvalues. The standardized BV were weighted with the corresponding eigenvector coefficients to construct an index that showed, in a single indicator, the most complete expression of the existing genetic variability in the animals’ ability to produce fpy along the trajectories of THI and DIM. This new option will make it easier to select animals which are more productive, and with better adaptability to heat stress, as well as enabling us to exploit genetic variations in the form of the response to heat stress to be adapted to different production systems.

## 1. Introduction

Although it is generally recognized that goats are more tolerant to heat stress (HS) than sheep and cows, due to the morphological and physiological differences between these species related to heat dissipation [1], various studies show the negative effect of HS on well-being and productivity in this species [2,3]. In several studies carried out to quantify these effects in various autochthonous Spanish breeds of goats, namely Murciano-Granadina and Payoya [4] Florida [5] and Malagueña [6], raised mostly in the South of Spain (the region of Spain most affected by heat), it was observed that the animals were exposed to stressful climatic conditions, due to the high temperatures, during 45 to 55% of the year, which generated losses of 1.9 and 3.1% of the annual fat plus protein yields (fpy) in Murciano-Granadina and Payoya goats [4], respectively. In addition, results published over the last 15 years [7], showed the importance of the genetic variation underlying such effects could be an extremely useful tool to facilitate the selection of animals more tolerant to HS.

Ravagnolo and Misztal [8] were the first to apply a random regression model (RRM) with test day (TD) production recorded monthly as a dependent variable and with a Temperature Humidity Index (THI) as a covariable, to estimate the genetic parameters and Breeding Values (BV) over the trajectory of the climatological variables. Since then, many studies have been published highlighting the existence of an antagonism between HS tolerance and milk production in cattle and small ruminant dairy animals, based on the negative value of the correlation between the intercept and the slope of the random regression model (RRM) used [9]. The general conclusion presented by Misztal [10] is that selection based only on milk production, without considering the HS, might result in animals which are more susceptible to heat stress. In other words, there is an interaction between milk production and HS tolerance.

The slope of the random regression model represents the animal’s ability to adapt and modify the expression of its genotype over the scale of environmental levels. This is known as the reaction norm (RN) and is the consequence of a biological process called plasticity, the usefulness of which in breeding programs has been highlighted by de Jong and Bijma [11]. In this scenario of HS effects on TD in dairy animals, two aspect of the results of the statistical models applied need to be considered further. Firstly, the results of the negative covariance between intercept and slope (cis) are only applicable along the THI trajectory, since they implicitly assume that such parameters do not vary on the days in milk (DIM) scale which was incorporated as a fixed covariate. Secondly, the DIM and THI trajectories have different characteristics and their results cannot to be treated in the same way. Milk production normally begins after calving and is expressed continuously along a time scale quantified by DIM. In contrast, the THI index represents a gradient of climatological intensity, but its trajectory fluctuates over the DIM, creating multiple interactions that can bias the estimation and interpretation of the cis, which supports the selection of animals tolerant to HS.

To find a solution to this problem, some authors [12,13] have studied the effects of HS on milk production by fitting the DIM and THI trajectories as random effects in the same model. These authors have shown that there is heterogeneity in the estimated genetic parameters, which further complicates the selection process since it is quite difficult to interpret the resulting complex longitudinal genetic (co)variance structure between both continuous variables. In that context, the use of a principal components analysis (PCA) could be a useful tool, allowing a significant reduction of the number of variables with a minimum loss of information [14]. Another advantage to the use of PCA is that the eigenvectors of the leading eigenvalues are mutually orthogonal, which can be used as a weighted factor in a new subjacent index. This procedure was presented by Togashi and Lin [15] and successfully used in different scenarios of animal breeding;Venturini et al. [16] for egg production; Boligon, et al. [17] in beef cattle; Macciota et al. [18] in dairy cattle.

The purpose of this study was to develop a different approach combining the results from RN and TDM models using principal components analysis to create a new subjacent variable that could provide a more robust and general indicator of HS tolerance for the daily fat plus protein yield (fpy) along the scales of THI and DIM.

## 2. Materials and Methods

For this study, the three Spanish goat breeds with the highest census and geographical distribution in Spain were selected. These are also the ones with the highest milk production. The Murciano Granadina goat breed (MG) has an average live weight of 50 kilos, a uniform black or mahogany coat and is generally exploited in intensive production systems throughout Spain. It is also exploited in other countries, generally in semistable systems. The Malagueña goat breed (Ma) has a live weight that ranges between 45 and 60 kilos, a uniform coat of blond color and is usually associated with pastoral or semistable systems. It is mainly distributed in southern Spain. Finally, the Florida goat breed ((Fl), of great strength and dairy structure, is characterized by its mottled coat and adapts to intensive production and semistall systems. It was distributed throughout all of Spain, and countries like Portugal, France and Italy (in a more or less testimonial way). These three breeds make up 90% of the census of the dairy goat in Spain (MG about 200,000 breeding goats, and Ma and Fl, 100,000 each). These three breeds present cheese extract (kilograms of fat + kilograms of protein) as the main selection criterion. 

In this study, we used the test day (TD) monthly records of fpy, expressed in total daily values, recorded from 2008 to 2018 through the regional milk recording scheme of these breeds, and supplied by the respective breeder’s societies (Acriflor: Asociación Nacional de Criadores de Ganado Caprino de Raza Florida; Acrimur: Asociación Española de Criadores de la Cabra Murciano-Granadina; Caprama: Asociación Española de Criadores de la Cabra Malagueña). Records outside the range of ±3 standard deviations, and any combinations of flock-control date with less than 10 observations, were deleted. Finally, a total number of 11,244 animals and 126,825 TD; 12,215 animals and 141,856 TD and 5162 animals and 62,834 TD from Fl, Ma and MG, respectively, were used in this study. The information for the pedigree was supplied by the respective breeding societies and covered only two generations back. These data sets were merged with climatological information from an area of no more than 15 km from the flock, provided by the Official Meteorological Agency of the Spanish Ministry for the Ecological Transition and the Demographic Challenge, consisting of the average values of maximum temperature (T in °C) and relative humidity (RH in %) recorded two days before the TD plus the day of the sampling. The results of T and RH were combined according to THI = [T − (0.55 × (1 − RH)) × (T − 14.4)] whose utility was showed by [19] for small ruminants in Mediterranean environmental conditions. General information of the analyzed dataset is presented in Table 1.

### 2.1. Statistical Procedure

The information from each breed was independently analyzed in two steps. First, estimation of the responses of the dependent variable (fpy) throughout the THI and DIM scales using linear fixed effects models, including contemporary group (flock-control date) and age at calving. The adjusted least square means for THI class effect were used in a fixed regression analysis to represent the form of response of fpy for each breed along this climatological indicator of HS. Accordingly, three groups of climatic conditions were created by direct inspection of these response along the THI trajectory: cold (THI ≤ 16); neutral (THI ≥ 17 to THI ≤ 25) and hot (THI ≥ 26). The distribution of the observations within each breed and climatological zone were proportionally similar. To estimate the effect of these climatological zones, a second linear fixed effects model, similar to the previous one, was applied, and the interaction of these three THI zone with DIM was included.

Second, two similar RRMs were applied to estimate genetic and individual permanent environment (co)variance components and the BV along two different trajectories: a time scale, represented by DIM, expressed in weeks, and another environmental scale quantified by the climatic index THI, both of which influenced the production of the animal’s fpy on each of the TD. The mixed linear RRMs were represented as follows:

**y = FTD_i_ + fixed_j_ + f(Φ _DIM:r_)_k_ + r(a; Φ _DIM:q_)_l_ + r(p; Φ _DIM:t_)_n_ + e_ijkln_ → M1**

**y = FTD_i_ + fixed_j_ + f(Φ _DIM:r_)_k_ + f(Φ _THI:r_)_l_ + r(a; Φ _THI:q_)_m_ + r(p; Φ _THI:t_)_n_ + e_ijklmno_ → M2**

where **y** represents the ith observation of the dependent variable (fpy) modeled along the **DIM** scaled by a repeatability animal model known as Test Day Model (TDM) in **M1**, and by a RN along the trajectory of THI in M2. **FTD_i_** is a fixed effect of the flock-control date combination, with 725, 637, and 474 levels for Fl, Ma and MG breeds, respectively. **Fixed_j_** are different fixed effects of jth age at calving (**j** = 1,2…9 years) in the three breeds. **f(Φ _DIM:r_)_k_** and **f(Φ _THI:r_)_l_** represent the fixed covariates for the kth DIM and lth THI scales respectively, modeled with a Legendre polynomial (**Φ**) of the order r = 3; **r(a;** **Φ _DIM__:q_)_l_,** and **r(a;** **Φ _THI i:q_)_m_** are vectors of random effects representing the additive genetic function of the animal (**a**) along the covariates **Φ _DIM:r_** or **Φ _THI__:r_** for kth DIM and lth THI respectively. In both cases modeled by a Legendre polynomial of order q, **r(p;** **Φ _DIM:t_)_n_** and **r(p;** **Φ _THI:t_)_n_** are random functions of permanent environmental effects (p) due to repetitions of the same data of the ith animal, modeled by a Legendre polynomial of order **t** and **e_ijkln_** and **e_ijklmno_** are random errors common to all observations.

The M1 model is the traditional TDM used at present in the breeding programs of these breeds to estimate the genetic (co)var components and breeding values (**BV**) as a tool to select animals to improve the level of **fpy** in these three goat breeds. M2, known as the RN model, allows us to estimate the (co)var components along the trajectory of **THI** in order to estimate the genetic components of adaptation to changes in the level of **HS** using two fixed covariates (**DIM** and **THI**). These models were independently applied to the data from each breed using ASReml 3 software (VSN international, Hemel Hempstead, UK) [20].

The main purpose of this work was not to compare statistical models, but rather to combine the (co)variance components and the BV of a trait, expressed simultaneously in the **DIM** and **THI** scales, to identify **HS** tolerance. Different orders (1 to 3) of the Legendre polynomial were tested but the best fit for our data sets was **q** = 2 and **t** = 1 for the animal and individual permanent environment effects in **TDM** and **RN**. For the Ka matrix the expected (co) variance components were:

$$V_{\left(y\right)}=A⊗K_{a}=\Phi_{i}\left[\begin{array}{ccc} \sigma_{a_{o}}^{2} & \sigma_{a_{os}} & \sigma_{a_{oq}}\\ \sigma_{a_{so}} & \sigma_{a_{s}}^{2} & \sigma_{a_{sq}}\\ \sigma_{a_{qo}} & \sigma_{a_{qs}} & \sigma_{a_{q}}^{2} \end{array}\right]\Phi_{i}^{\prime }+I_{p}⊗K_{p}=\Phi_{i}\left[\begin{array}{cc} \sigma_{p_{o}}^{2} & \sigma_{p_{os}}\\ \sigma_{p_{so}} & \sigma_{p_{s}}^{2} \end{array}\right]\Phi_{i}^{\prime }+R$$

The elements of **Φ** are the coefficients of the Legendre polynomial, expressed in a standardized way between +1 and −1 for the THI or DIM trajectories. In these models, the structure of (co)variance **Ka** contains elements of the genetic function for the intercept ($\sigma_{a_{o}}^{2}$), slope ($\sigma_{a_{s}}^{2}$) and for the quadratic term ($\sigma_{a_{q}}^{2}$) and their respective covariances ($\sigma_{a}{}_{{}_{os}};\sigma_{a}{{}_{{}_{oq}}}_{}^{}\;\mathrm{and}\;\sigma_{a}{}_{{}_{sq}}$) throughout **THI** or **DIM**. On the other hand, **Kp** contains the same elements for the (co)variances ($\sigma_{p_{o}}^{2}$**;** $\sigma_{p_{s}}^{2}$ and $\sigma_{p_{os}}$) for permanent individual environmental effects, with **Ip** as the incidence matrix for this effect in all three models; **R** is the homogeneous residual variance; **A** is the numerator of the relationship matrix and ⊗ is the Kronecker product indicator. With the results of these models, it is possible to estimate the heritability (h^2^) and the genetic correlations (r_g_) for fpy at each point of the THI or DIM scales using the corresponding coefficients of **Φ** according to the procedure presented by Jamrozik and Schaeffer [21] and shown in the Appendix A.

### 2.2. Breeding Value Estimation for THI and DIM and Development of a New Index

With the solutions of these models, the vectors of the genetic functions for gf_DIM_ = [a_o_ a_s_ a_q_] and for gf_THI_ = [a_o_ a_s_ a_q_] were used to estimate the BV for fpy along the scales of DIM and THI, respectively, in which the elements a_o_; a_s_ and a_q_ represent the genetic merit of each animal for such components. The genetic functions gf_THI_ and gf_DIM_ were estimated with the same data, and their elements are considered as properties of each animal’s BV, which can be expressed for each point of the **THI** or **DIM** trajectories, as shown in the Appendix A. These BV were subjected to a PCA score option of Matlab (The Math Works, Inc, Natick, USA) [22] and the corresponding eigenvectors (ev) coefficients of the leading eigenvalues were used as weighting factors in a new underlying index (Ipc) following the detailed procedure presented by Togashi and Lin [15]:

$$Ipc=ev_{1}^{\prime }*BV_{z}+v_{2}^{\prime }*BV_{z}$$

where **ev_1_** and **ev_2_** are the **ev** of two first leading eigenvalues and **BV_z_** are the Breeding Values of each animal expressed in a standardized way

## 3. Results

### 3.1. Overall Results

**Figure 1:**
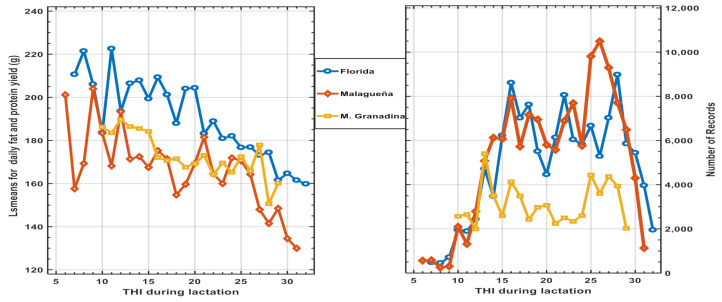
Least square means and distribution of records showing the effect of temperature and relative humidity index (**THI**) level during lactation on total daily fat and protein yield in three Spanish dairy goat breeds.

The results of the previously defined fixed effects models were highly significant (*p* < 0.001) in each of the three breeds studied. The least square means for **THI** effects for each breed are presented in Figure 1, as well as the distribution of the number of records available.

The differences between the three climatic zones were highly significant (*p* < 0.001), but more evident in the FL and MG breeds, although similar trends of decreasing fpy throughout lactation are presented. The regression analysis within breed for fpy on THI level, weighted by the number of observations, showed that all the regression coefficients (b) were negative and highly significant (*p* < 0.001): b = −2.367 (±0.255)/g/THI, for Fl; b = −0.589 (±0.222)/g/THI, for Ma and for MG b = −1.332 (±0.258)/gm/THI. These regression estimates indicate a phenotype antagonism between HS and fpy throughout the trajectory THI (Table 2). The total impact of the effect of THI was equivalent to 13.5 to 30 days in production in these dairy goat breeds.

**Figure 2:**
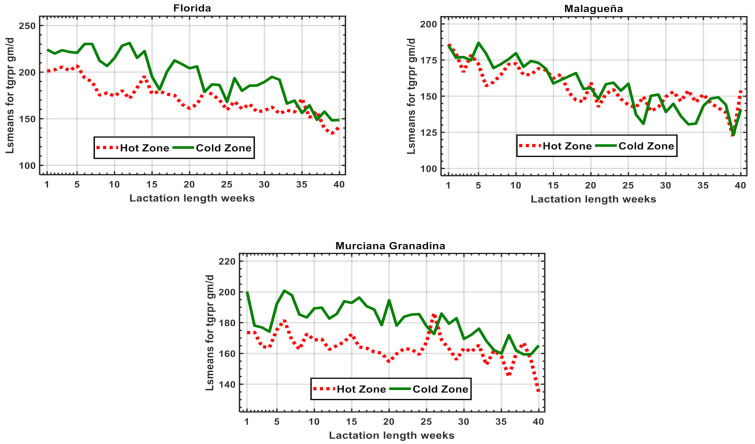
Least squares mean for total daily fat and protein yield (tgrpr) throughout lactation and two extreme climatic zones.

The adjusted least square means for each breed, showing the results of the first 280 days of lactation in two extreme climatic zones (hot and cold) are shown in Figure 2.

### 3.2. Results of Genetic Models along DIM. (Model 1)

**Figure 3:**
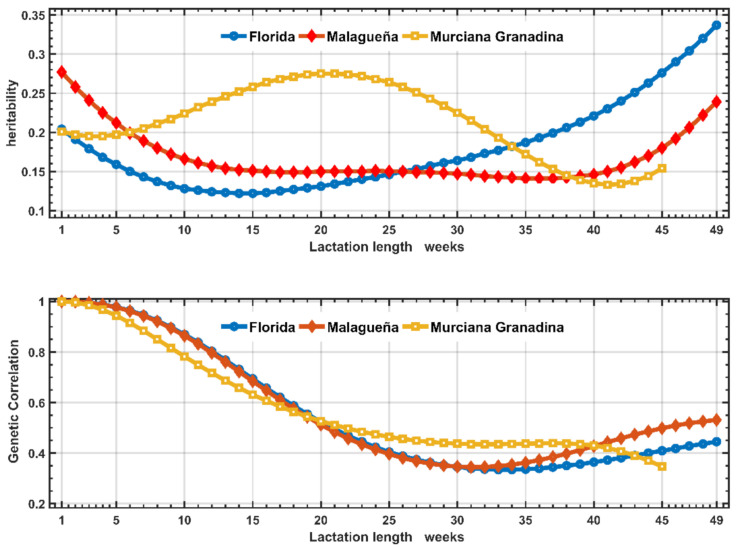
Estimates of heritability and genetic correlations between the first week of lactation and the rest of lactation obtained with the classic Test Day Model (model 1) in three Spanish breeds of goats.

The genetic parameters for fpy throughout the DIM scale estimated by Model 1 are presented in Figure 3, in which it is evident that h^2^ manifested the same pattern during the first 30 weeks for FL and Ma, while for MG the result was different, with an increased h^2^ value from the first week up to week 25 and decreasing later. The genetic correlations showed the same results for the three breeds, with values that decreased as the differences between the first week and the rest of **DIM** increased.

The results of PCA of the Ka genetic matrix from TDM model 1 (Table 3) show that the first principal component (PC1) explains most of the level of genetic variation for the trait across lactation length, which can be modified by PC2 and PC3, which together explain between 17 and 25% of the genetic variance for fpy. Even though the corresponding eigenvectors are mutually orthogonal, there are some differences between each breed. In the context of the objective of this study these results could be useful for breeding as presented later in the article.

### 3.3. Results of Genetic Models along THI Effects (Model 2)

The Ka matrices from model 2 for each breed were subject to a PCA analysis, and the results are presented in Table 4. Note that the quadratic terms of this model for the Malagueña breed are not presented here because it was not possible to obtain convergence. The two first components (PC) explained between 98 and 100% of the genetic variance of fpy along the THI scale. In the three breeds, PC1 explained between 92 and 93% of the general level of genetic variance of fpy, which is the intercept of the RN, and the relative importance of PC2 and PC3 was between 6.7 to 7.2%

**Figure 4:**
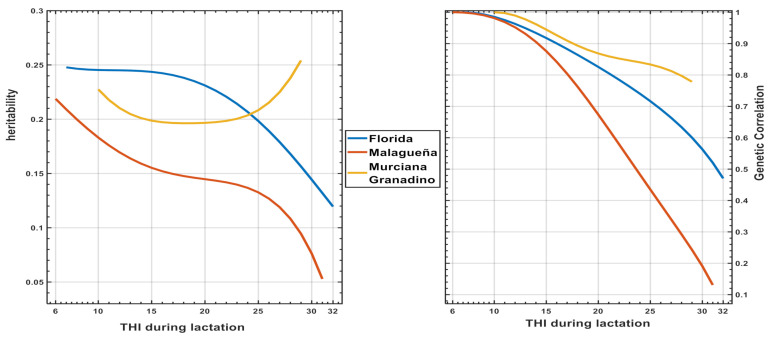
Variation along the THI trajectory during lactation in the estimates of heritability and genetic correlations for total fat and protein production yield obtained with RN model 2 in three Spanish dairy goats breeds.

Figure 4 shows the genetic parameters estimated by **RN** model 2. The values of h^2^ and the genetic correlation decrease as the **THI** level increases, except the value of h^2^ for MG. The results of the three breeds are coherent with the existence of an important genetic variability across the trajectory of THI, but the relationship between fpy at different level of HS is far from the unity, particularly for Fl and Ma.

### 3.4. Generalizing the Response Forms of Fat Plus Protein Yield along the Trajectories of THI and DIM and Construction of the New Index

**Figure 5:**
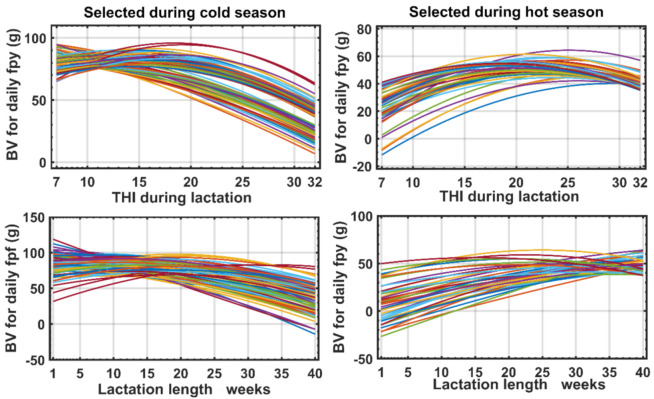
Variation in the form of response of the Breeding Values (BV) throughout the weeks of lactation and THI levels for daily fat and protein yield (g) estimated from the best 500 animals in the Florida breed.

As an example of the importance of previous results we selected the best 500 animals from the Florida goat breed, according to their original BV for **fpy** both in the cold zone and in the hot zone, as previously defined (the results Ma and MG were similar but in Supplemental Figures S1 and S2 more general informative figure are presented). Figure 5 shows the evolution of the BV for a large group of these selected animals for each point of **THI** (upper part of Figure 5). Only 63% of the animals were the best in both cold and hot zones, while the BV of animals selected in the cold season decreased along THI, but the response of the best animals selected in the hot season ed the opposite direction. Interesting results were observed for the response of the same animals selected in the cold and hot seasons, but along the trajectory of DIM (lower part of Figure 5), which follows the same previous pattern.

To generalize the relationships between HS and fpy production, we need to consider that the solution of the TDM and RN models provides the genetic merits of each animal which are expressed in terms of the genetic functions (gf) along the scales of DIM and THI. These gfs are made up of three elements: an intercept; a slope and a quadratic term, which are inseparable parts of the genotype of each animal and need to be combined with the coefficients of the Legendre polynomial to estimate the BV along both the **DIM** and **THI** trajectories, respectively. Table 5 shows the correlations between the components of both genetic functions for each breed. The results indicate that the intercepts reveal a positive relationship in the three breeds, while the correlations between the intercepts and slopes are negative in correspondence with the previous pattern.

**Figure 6:**
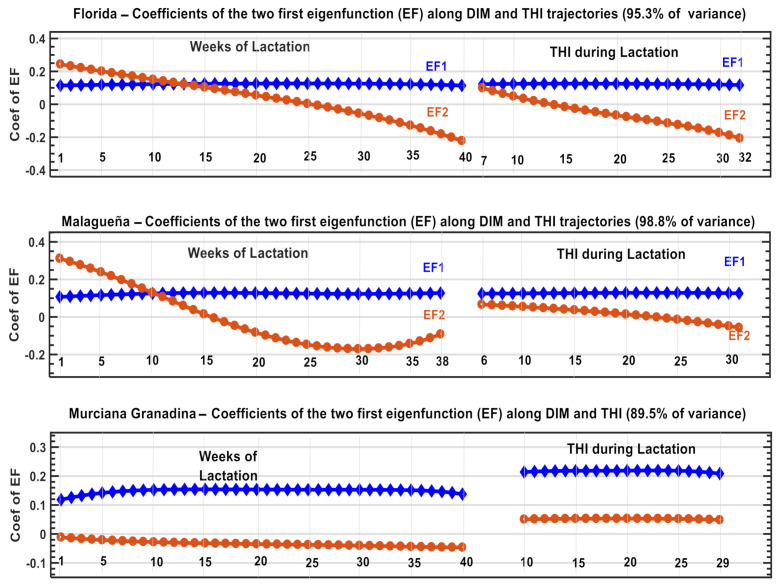
Eigenvector coefficients and percentage of variance explained by the two first Principal Components of Breeding Values estimated for different points of the trajectories of THI and week of lactation in three dairy goat breeds in Spain.

To examine the possibility of attenuating the effect of the interaction between THI and DIM, we used the BV for the daily fpy during the first 40 weeks of lactation, since it was representative of the DIM trajectory (model M1) and of each level of THI (model M2). The resulting new dataset contains the information of 66, 64 and 65 estimations of BV for Fl and Ma and MG, respectively, which contain all the variability and form of response of fpy in both scales, which were standardized and subjected to a PCA analysis. The results are illustrated in Figure 6, showing that the two first PCs explain between 89% and 98% of the total variance. The corresponding eigenvector coefficients, which constitute the eigenfunction of the PCA, synthesize the magnitude and direction of the (co)variances between the BV across THI and DIM. The coefficients of PC1 present positive values and a similar contribution to each expression of BV in the three breeds. The relative importance and the direction of the eigenvectors of PC2 showed differences between breeds. For Fl and Ma, the coefficients decreased as THI levels and DIM increases, whereas for MG, the pattern is different.

**Figure 7:**
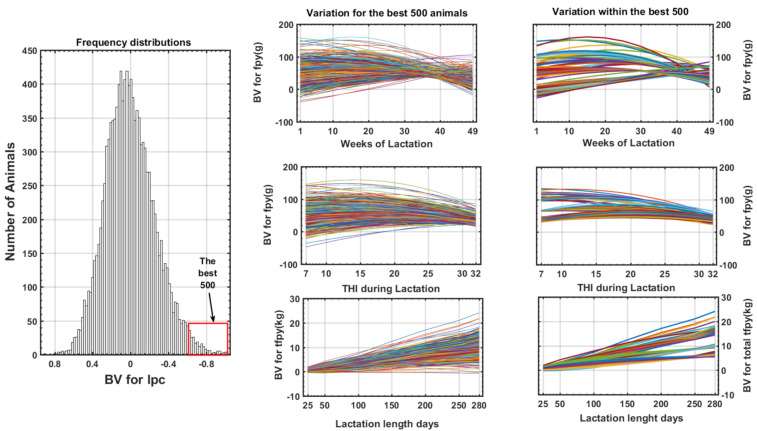
Frequency distributions of the new Ipc index (**left**) for Florida goats and variability of the best 500 animals for daily fpy (**right**) along DIM and THI and cumulate fpy at different point of lactation length.

Previous results show that with only two eigenvector it is possible to examine a new alternative for a solution of the complex genetic (co)variance structures for fpy along the scale of THI and DIM using the new index (Ipc) presented in the Material and Methods section. In that sense we selected the best 500 animals according to Ipc, and the evolution of the original BV during the cold and hot season are presented in Figure 7 for Florida goat (Supplemental Figures S1 and S2) for Malagueña and Murciana Granadina goats, respectively. The correlation with Ipc with the original BV in the cold, neutral and hot seasons were 0.986; 0.958 and 0.857 respectively and 88.4% of the best animals in the cold zone were the best in hot season. The use of Ipc allows us to obtain the same type of response along the THI or DIM scale for the best animals selected in the cold or hot season which is the opposite of that observed in Figure 4. All results of the use of Ipc allow us to identify those animals with a higher HS tolerance without compromising the genetic level of daily fpy during the lactation length.

## 4. Discussion

The antagonism between HS and fpy (Figure 2) and its possible economic impact (Table 2) found in this study, is consistent with the results reported in previous publications on these breeds [5]. In the future, this problem could have a huge impact on the sector in Spain, since most goat farms are located in central and southern parts of the country, regions where the temperature has increased almost twice as much as the European average [23].

Carabaño et al. [9] showed that there is a negative relationship between the intercept and the slope of the reaction norm models, and we found similar results in this study (see Table 5) in three goat breeds in Spain. Even if results for MG were slightly different in some parameters, the general pattern presented in Supplemental Figure S1 is the same as for Fl and Ma. Since the first published results on this subject [8], an RN model has been applied, which included two fixed covariates: one linked to DIM and another to the THI level on the same date of the test day records. This methodology worked very well and has been applied in a number of studies [24,25], which all concur in highlighting the genotype environmental interaction (Ige) between DIM and THI. However, the nature of such Ige effects has rarely been studied in a trait expressed simultaneously along the trajectories of time (DIM) interacting arbitrarily with the environmental variable (THI level).

In the context of the RRM model applied, the results were expressed in terms of a gf. In our study an order r = 2 was used and, therefore, these gfs were made up of three elements with very precise biological meaning: an intercept (**a_o_**) that defines the animal’s general genetic level for production, a slope (**a_s_**) defining the animal’s ability to react and change the level of production, and a quadratic term (**a_q_**) that modifies (accelerating or decreasing) the effect of the slope. Our results in Table 5 show a positive correlation between **a_o_** for the three breeds, meaning that if the selection process favored these components the genetic level of the animal increases along the scales of DIM and THI, but at the same time the correlated response is negative due to an antagonist correlation between a_o_ and a_s._ As a consequence, the results suggest more productive animals but with less adaptation to change in THI, which is an expression of Ige in line with the same general conclusions presented by Misztal [10]. The results of the genetic correlations between the fpy at different levels of THI or DIM trajectories found in this study (Figure 3 and Figure 4) agree with previous studies showing the same decreasing pattern for dairy traits in goats [26]; sheep [5,19] and dairy cattle [9,27] and cannot be considered as the same traits across both scales.

In the previously referenced articles, DIM and THI were fixed covariates in the RN models. Therefore, it was assumed, a priori, that there were no variations between the animals in their ability to adapt to changes in THI throughout the trajectory of DIM. However, several articles [12,28,29] showed the existence of heterogeneity in the (co)variance estimates along THI and DIM. Our study agrees with these latter, showing the nature of these Ige interactions expressed at two levels affecting the daily fpy along DIM (Model 1, Figure 3) and THI trajectories (Model 2, Figure 4). The results show the existence of a double Ige (DIge): the first related to change in BV ranking of the animals across both scales, and the second connected to the differences in BV in the type of response of the daily production of fpy along DIM and THI (Figure 3 and Figure 4). The latter constitutes a serious limiting factor for the selection of more productive animals, which are at the same time adapted to HS. The PCA used in our study can be a useful tool to attenuate this limitation, using the BV of the animals from the TDM and RN models (Models 1 and 2, respectively).

The approaches applied in this article are based on the definition given by Stinchcombe and Kirkpatrick [30] for those traits whose responses are continuous functions of other variables, denoted by these authors as ‘function value traits’ (fvt). This is the case for fpy, which depends on the joint variations of the trajectories of two continuous variables. We examined this alternative using the global analysis of the BV estimated for each animal along the trajectories of DIM and THI, which represents the most complete expression of the genetic variability existing in the animals’ capacity for the production of fpy, whatever the combinations of both variables. We can combine these BV through a PCA, using the corresponding eigenvector of the leading PC as a weighting factor in an index (Ipc). This was originally proposed by Togashi and Lin [31] and has been recently successfully applied to 19 traits related to egg production [31], and on 11 growth and reproduction traits in Brazilian beef cattle [17]. Hammami et al. [32] and Carabaño et al. [33] presented an approach closely related to ours, for using the estimations of BV for THI in different countries, and Macciotta et al. [18] based their study on the intercept and slope of the RN.

The results obtained with this PCA (Figure 6) indicate that the first two eigenvectors (**ev_i_**) explain almost all the variances between the estimates of the animals’ BV throughout the scales of DIM and THI in the three goat breeds. Since the ev_i_ are orthogonal, we can add their effects in a single Ipc index, which considerably reduces the number of variables, retaining most of the (co)variance of each one. These ev_i_ values are the coefficients of the eigenfunction (ef_i_) for the fvt traits defined by Kirkpatrick and Meyer [34] and provide the basis of this Ipc, which can improve the selection process considerably.

Druet et al. [35] applied the same approach when studying monthly milk production records, considering **ef_1_** as an indicator of the general genetic level and **ef_2_** as the persistence of lactation. These authors highlighted the usefulness of **ef_i_** in the selection process of Holstein cattle in France. The trends of the coefficients presented in (Figure 6) follow the same pattern but related to the production of fpy throughout the scale of DIM and THI level. The selection of the best 500 animals according to Ipc is consistent with this point of view, and we have shown (Figure 7 and Supplemental Figures S1 and S2) that it is possible to identify the animals with the highest genetic merits across both trajectories. The use of the results from the Ipc allow us to attenuate the restricted effect of DIge presented in this study and, at the same time, we can exploit the additional information related to genetic variations in the type of response in order to identify the animals best adapted to the different production systems.

## 5. Conclusions

The current study, in the three main autochthonous dairy goat breeds from Spain, corroborates the existence of dairy production and heat stress antagonism, which has already been published in different species. Our results show an important (co)variance genetic variability for daily production of fpy in the three breeds, with a depressive pattern along the scale of DIM and THI, which means that they are not the same traits along the trajectories of both continue variables. The level of fpy is subject to a double genotype-environment interaction (DIge), expressing its effect at the level of DIM and THI trajectories, which represent a major obstacle to the breeding program because both effects act together during lactation of progenies of the best selected parents. The breeding value (BV) for all the animals of each breed, along the scales of time (DIM) and heat stress (THI), were subjected to a PCA and the corresponding eigenvector of the leading eigenvalues were used as a weighting factor to combine the standardized BV in an index (Ipc), which allowed us to identify the most tolerant animals to HS without negatively affecting the genetic potential for milk production. We would recommend the use of this Ipc approach to attenuate the effects of this DIge.

## Figures and Tables

**Table 1:** Population parameters of the three analyzed dairy goat breeds.

	Breeds		
	Florida	Malagueña	Murciano-Granadina
**Number of records**	126,825	141,856	62,834
**Number of animals**	11,244	12,215	5162
**Number of dams**	1999	2650	2198
**Number of sires**	233	158	215
**Number of flocks**	20	17	20
**Animals in pedigree**	12,268	14,075	6037
**THI ^1^ level**	21.4 (5.9) ^2^	21.4 (5.7)	19.6 (5.8)
**N^o^ of recorded flock-date**	725	637	474
**Average days in milk**	122.6 (72.8)	131.3 (77.3)	130.1 (76.7)
**Daily fat and protein yield (g)**	190.7 (83.5)	162.1 (70.9)	176.4 (80.5)

^1^ THI, combined index for temperature (in °C) and relative humidity (in %). ^2^ Standard Deviation in parentheses.

**Table 2:** Indicators of the effects of three THI^*^ zones throughout lactation in three Spanish of dairy goat breeds.

	Florida			Malagueña			Murciana-Granadina		
	Cold	Neutral	Hot	Cold	Neutral	Hot	Cold	Neutral	Hot
**%Time of the year**	24.4	45.2	30.4	24.3	44.6	30.9	36.3	34.3	29.2
**LSmeans in g**	194.5_(9.8)_	201.9_(3.8)_	170.8_(4.6)_	167.2_(0.6)_	163.8_(0.4)_	159.4_(0.6)_	176.7_(0.7)_	187.8_(0.73)_	180.9_(0.8)_
Regr-cold **	Y_280_ = 240.9 − 0.38 + 0.0004, R^2^ = 75.9%			Y_280_ = 196.1 − 0.36 + 0.00008, R^2^ = 63.1%			Y_280_ = 194.9 − 0.09, R^2^ = 20.0%		
Regr-hot ***	Y_280_ = 209.5 − 0.24, R^2^ = 83.2%			Y_280_ = 186.0 − 0.25 + 0.00008, R^2^ = 67.0%			Y_280_ = 179.9 − 0.06, R^2^ = 17.1%		
Tot fpy **** hot	47.3 kg			42.1 kg			46.9 kg		
Tot fpy **** cold	53.0 kg			44.3 kg			50.2 kg		
**Differences**	−5.8 kg			−2.2 kg			−3.3 kg		
**Equivalent in days**	30			13.5			18.8		

Standard error in parentheses. THI, combined index for temperature and relative humidity; Cold THI ≤ 16 and Hot THI ≥ 26. ** Regression equation from the least squares means (LSmeans) related to weeks of lactation. *** THI zone weighted by the number of records in each week of lactation. **** Total daily protein and fat yield (fpy) estimated as the sum of the LSmeans within each THI zone from 10 to 280 days of lactation.

**Table 3:** Elements of the genetic random regression model 1; eigenvalues and eigenvector coefficients for the first three Principal Components (PC) from Ka matrix (Model 1) for daily fat protein yield in the three Spanish goat breeds studied.

	Florida			Malagueña			Murciano−Granadina		
	PC1	PC2	PC3	PC1	PC2	PC3	PC1	PC2	PC3
**Intercept**	0.990	0.126	−0.052	0.9814	0.1905	0.0245	−0.974	0.131	0.183
**Slope**	0.133	−0.975	0.178	−0.1911	0.9564	0.2210	0.111	0.987	−0.117
**Quadratic**	0.028	0.183	0.983	0.0187	−0.2216	0.9750	0.196	0.094	0.976
**Eigenvalues, %**	78.5	14.7	6.8	74.9	15.5	9.6	83.6	9.5	6.9
**Cumulative Variance explained**		93.2	100		89.7	100		93.1	100

**Table 4:** Elements of the genetic random regression model 2; eigenvalues and eigenvector coefficients for the first three Principal Components (PC) from Ka matrix (Model 2) for daily fat protein yield in the three Spanish goat breeds studied.

	Florida			Malagueña		Murciano-Granadina		
	PC1	PC2	PC3	PC1	PC2	PC1	PC2	PC3
**Intercept**	−0.9875	0.1187	0.1037	0.9853	0.1711	0.9991	0.0212	0.0356
**Slope**	0.1356	0.9752	0.1750	−0.1711	0.9853	−0.0265	0.9874	0.1562
**Quadratic**	0.0804	−0.1869	0.9791	na	na	0.0318	0.1571	−0.9871
**Eigenvalues %**	92.70	7.20	0.10	92.77	7.23	93.30	4.85	1.85
**Cumulative Variance explained**		99.9	100		100		98.15	100

**Table 5:** Correlation between the Breeding Value for the genetic function elements * estimated along the trajectory of **DIM** or **THI** in the three Spanish goat breeds.

	Florida			Malagueña			Murciano-Granadina		
	a_o_THI	a_s_THI	a_q_THI	a_o_THI	a_s_THI	a_q_THI	a_o_THI	a_s_THI	a_q_THI
**a_o_DIM**	0.880	−0.418	−0.252	0.968	−0.389	X	0.950	−0.489	−0.527
**a_s_DIM**	−0.497	0.351	0.021	−0.483	0.401	X	−0.182	0.155	0.177
**a_q_DIM**	−0.905	0.403	0.292	0.068	−0.302	X	0.225	−0.153	−0.040

* **a_o_THI, a_s_THI, a_q_THI** are the intercept, slope and quadratic term for **THI** and **a_o_DIM, a_s_DIM, a_q_DIM** are the intercept, slope and quadratic term for **DIM.**

## Data Availability

Not applicable.

## Supplementary Materials

The following are available online at https://www.mdpi.com/2076-2615/11/3/736/s1, Figure S1. Frequency distributions of the new Ipc index (left) for Malagueña goat and variability of the best 500 animals for daily fat and protein yield (fpy) along DIM and THI and total cumulate fpy (tfpy) (right) at different point of lactation length. Figure S2. Frequency distributions of the new Ipc index (left) for Murciana Granadina goat and variability of the best 500 animals for daily fat and protein yield (fpy) along DIM and THI and total cumulate fpy (tfpy) (right) at different point of lactation length.

## Appendix A

This additional document presents, step by step, the procedure used to estimate the genetic (co)variance components and the Breeding Values (BV) for total fat and protein production throughout the **THI** scale, according to the Reaction Norm Model (model 2).

### Heritability, Genetic Correlation and Breeding Values

The basic elements are contained in the random regression matrices of genetic origin (**Ka**) of order **r** = 2 and those of individual permanent environment (**Kp**) of order **t** = 1, as well as the corresponding coefficients of the Legendre polynomial. These coefficients have three elements, one for the intercept, another for the slope and the other for the quadratic term, and they are presented in Table S1 for three contrasting regions of the **THI** scale: Cold zone (**C**) with **THI** ≤ 16; Neutral zone (**N**) with **THI** = 17 to THI = 25 and Hot zone (**H**) with **THI** ≥ 26.
